# Conjugates of Phthalocyanines With Oligonucleotides as Reagents for Sensitized or Catalytic DNA Modification

**DOI:** 10.1155/BCA/2006/63703

**Published:** 2006-02-19

**Authors:** Alexander A. Chernonosov, Vladimir V. Koval, Dmitrii G. Knorre, Alexander A. Chernenko, Valentina M. Derkacheva, Eugenii A. Lukyanets, Olga S. Fedorova

**Affiliations:** ^1^Institute of Chemical Biology and Fundamental Medicine, Siberian Division of the Russian Academy of Sciences, Lavrentyev Avenue 8, Novosibirsk 630090, Russia; ^2^Novosibirsk State University, Pyrogova Street 2, Novosibirsk 630090, Russia; ^3^Institute of Human Ecology, Sovetskii Avenue 18, Kemerovo 650099, Russia; ^4^Institute of Semiconductor Physics, Siberian Division of the Russian Academy of Sciences, Lavrentyev Avenue 13, Novosibirsk 630090, Russia; ^5^Organic Intermediates and Dyes Institute, B. Sadovaya 1/4, Moscow 103787, Russia

## Abstract

Several conjugates of metallophthalocyanines with deoxyribooligonucleotides were synthesized to investigate sequence-specific modification of DNA by them. Oligonucleotide parts of these conjugates were responsible for the recognition of selected complementary sequences on the DNA target. Metallophthalocyanines were able to induce the DNA modification: phthalocyanines of Zn(II) and Al(III) were active as photosensitizers in the generation of singlet oxygen ^1^O_2_, while phthalocyanine of Co(II) promoted DNA oxidation by molecular oxygen through the catalysis of formation of reactive oxygen species (^.^O_2_^−^, H_2_O_2_, OH). Irradiation of the reaction mixture containing either Zn(II)- or Al(III)-tetracarboxyphthalocyanine conjugates of oligonucleotide pd(TCTTCCCA) with light of > 340 nm wavelength (Hg lamp or He/Ne laser) resulted in the modification of the 22-nucleotide target d(TGAATGGGAAGAGGGTCAGGTT). A conjugate of Co(II)-tetracarboxyphthalocyanine with the oligonucleotide was found to modify the DNA target in the presence of O_2_ and 2-mercaptoethanol or in the presence of H_2_O_2_. Under both sensitized and catalyzed conditions, the nucleotides G^13^–G^15^ were mainly modified, providing evidence that the reaction proceeded in the double-stranded oligonucleotide. These results suggest the possible use of phthalocyanine-oligonucleotide conjugates as novel artificial regulators of gene expression and therapeutic agents for treatment of cancer.

## INTRODUCTION

Design of sequence-specific DNA- or RNA-modifying reagents requires conjugation of recognition and reactive groups in a single molecule. Oligonucleotides, which are fragments of single-stranded RNA or DNA, possess an inherent ability to anneal to the respective complementary sequences of the nucleic acids inside cells. This specific interaction results in the formation of double-stranded complexes and provides a recognition mechanism necessary for copying and correcting genetic material. Because of their unique targeting ability, oligonucleotides are of particular interest in chemotherapy. They can serve as specific tags for nucleic-acid-damaging drugs. Inherently, nonspecific chemical compounds can be converted into accurate weapons when coupled to oligonucleotides. The reactive derivatives of oligonucleotides represent a wide class of specific reagents proposed for regulating gene expression (for a review, see [1]). This approach, relying on sequence-specific targeting of reactive compounds, was initially called “complementary addressed modification of nucleic acids” [2]. In its initial implementation, it was suitable for the modification of single stranded polynucleotide chains. Later, the approach was expanded to include nonreactive oligonucleotides and their synthetic analogs, forming the basis of the “antisense” technology (reviewed in [3]). It was also shown that the same method may be used for certain double-stranded nucleic acids capable of forming triple-stranded complexes (“antigene” approach) [4–6]. Presently, the methodology is considered universally applicable to suppress the development of viral infections as well as tumors induced by mutations in protooncogenes responsible for malignant cell transformation.

Various chemical groups were studied as reactive moieties in oligonucleotide conjugates [1]. Phthalocyanines have a number of chemical properties making them effective reagents for nucleic acid modification. They can both act as good photosensitizers, promoting singlet molecular oxygen formation, and effectively catalyze oxidation of organic molecules by molecular oxygen in dark. The presence of bridging nitrogen atoms in these compounds results in efficient absorption at 600–700 nm, making them sensitive to the light of this wavelength, which can penetrate deep into living tissues [7]. This property of phthalocyanines is used in photodynamic therapy (PDT) of cancer [8]. Singlet molecular oxygen ^1^O_2_ is active towards different cell components (nucleic acids, proteins, lipids) [9].

On the other hand, phthalocyanine complexes with some paramagnetic metal ions are able to catalyze formation of reactive oxygen species (ROS) such as superoxide radical, hydrogen peroxide, and hydroxyl radicals via reductive-oxidative mechanism. These species damage different biomolecules [10]. The complex of phthalocyanine with Co(II) is known to be one of the most effective catalysts in the oxidation of different substrates with molecular oxygen and hydrogen peroxide [11].

**Figure 1:**
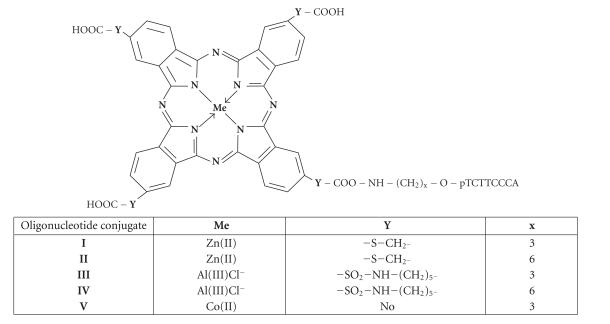
Structures of the phthalocyanine conjugates.

The main goal of the present work is to address the possibility of the modification of DNA by phthalocyanines of Al(III) and Zn(II) as sensitizers and Co(II)-phthalocyanine as a catalytic group conjugated to oligonucleotides. The structures of metallophthalocyanine conjugates are presented in Figure 1.

## EXPERIMENTAL METHODS

### Chemicals and reagents

Acrylamide, N,N′-methylene-bisacrylamide, urea, acetonitrile, DMF (Fluka, Switzerland), 1,3-diaminopropane (Merck, Germany), Tris-HCl, and 2-mercaptoethanol (Sigma, USA) were used. All buffer solutions were prepared with double-distilled water using ultrapure reagents. *E coli* 8-oxoguanine-DNA glycosylase was a courtesy of Dr A A Ishchenko, (ICBFM, Novosibirsk, Russia). All phthalocyanines investigated contained four carboxyl groups, which were used for conjugation with amino groups of the linkers attached to the 5′-terminal phosphate of the octanucleotide (8-nt).

### Oligonucleotides and conjugates

**Figure 2:**
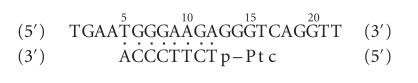
Sequence of the oligonucleotide duplex used for the complementary-addressed DNA modification. Ptc, tetra-4-carboxyphtalocyanine of Co(II), Al(III), or Zn(II), attached to the 5′-terminal phosphate of the addressing oligonucleotide *via* aminopropanol or aminohexanol linkers.

The 8-nt and 22-nt deoxyribonucleotides 5′-pdTCTTCCCA-3′ and 5′-dTGAATGGGAAGAGGGTCAGGTT-3′ (Figure 2) were synthesized on an ASM-700 automated synthesizer (BIOSSET Ltd., Novosibirsk, Russia) from phosphoramidites purchased from Glen Research (Sterling, Va, USA) according to the manufacturer's protocol. The oligonucleotides were deprotected with ammonium hydroxide and purified by ion exchange HPLC on a Nucleosil 100-10 N(CH_3_)_2_ column followed by reverse-phase HPLC on a Nucleosil 100-7 C_18_ column (both 4.6 × 250 mm, purchased from Macherey-Nagel, Düren, Germany). The purity of oligonucleotides exceeded 98%, as estimated by electrophoresis in 20% denaturing polyacrylamide gel after staining with Stains-All dye (Sigma-Aldrich, St Louis, Mo, USA). Concentrations of oligonucleotides were determined from their absorbance at 260 nm [12]. The 22-nt oligonucleotide was used as a DNA target; the 8-nt oligonucleotide was used for the synthesis of conjugates with phthalocyanines.

The oligonucleotide conjugates with phthalocyanine complexes of Al(III), Zn(II), and Co(II) were synthesized using a previously reported solid-phase method [13] with a 30–40% yield. The conjugates were isolated from the reaction mixture and purified by HPLC in an acetonitrile/water gradient. Reverse-phase HPLC profiles with different retention times for the free oligonucleotide and for the phthalocyanine-oligonucleotide conjugates provided a strong proof that the phthalocyanine moiety was covalently attached to the oligonucleotide [13].

### 5′-terminal phosphorylation

The 22-nt oligonucleotide was labeled with ^32^P at the 5′-end in a 30 *μ*L reaction mixture containing 30 pmol of the oligonucleotide, 30 pmol of [*γ*−^32^P]ATP (specific activity 3.3 × 10^−3^ mCi/pmol), and 10–20 units of T4 polynucleotide kinase (Sibenzyme, Russia) in a buffer containing 0.05 M imidazolium chloride (pH 6.6), 0.01 M MgCl_2_, 5 mM dithiothreitol, 0.1 mM spermidine, 0.5 mM ADP, and 0.1 mM EDTA. The mixture was incubated at 37°C for 30 minutes. The product was precipitated by adding 10 volumes of 2% LiClO_4_ in acetone, and further purified by electrophoresis in denaturing 20% polyacrylamide gel (PAGE). The oligonucleotide was electroeluted onto a DE-81 filter (Whatman, Brentford, UK), stripped from the filter with 3 M LiClO_4_ at 56°C, and precipitated with 2% LiClO_4_ in acetone. The precipitate was washed with acetone (3 × 200 *μ*L), dried in vacuum, and dissolved in double-distilled water (100 *μ*L). The concentration of the resulting solution of the labeled oligonucleotide did not exceed 2.5 × 10^−7^ M.

### Modification

All modification was carried out in a buffer containing 0.16 M NaCl, 0.02 M sodium phosphate (pH 7.4), and 1 mM EDTA.

#### Sensitized modification

**Figure 3:**
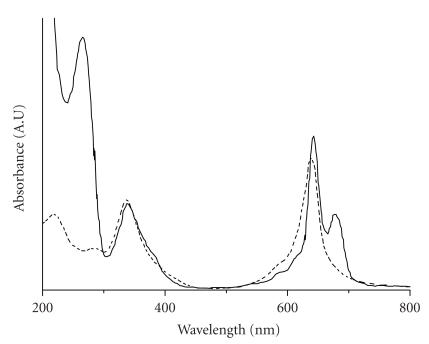
Absorption spectra of aqueous solutions of Al(III) tetra-[4-carboxypentenylsulfamoyl]-phthalocyanine (dashed line) and its conjugate with d(pTCTTCCCA) (solid line).

**Figure 4:**
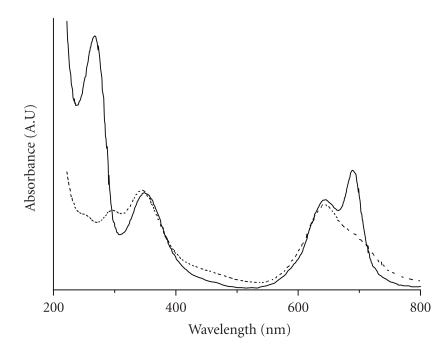
Absorption spectra of aqueous solution of Zn(II) tetra-4-carboxymethylthiophtalocyanin (dashed line) and its conjugates with d(pTCTTCCCA) (solid line).

The absorption spectra of phthalocyanines contain the Soret band at about 340 nm and two Q-bands at 630 nm and 680 nm (Figures 3 and 4). Therefore, the reactive moiety of the conjugates can be excited at different wavelengths using light of a high-pressure mercury lamp (*λ*;_max_ = 365 nm) or a helium-neon laser (*λ*;_max_ = 633 nm), respectively.

Irradiation of the samples at 365 nm was carried out for 3 hours in a photochemical reactor equipped with a quartz rectangular cell of 0.1 cm path length. This cell was immersed in a cuvette filled with the aqueous solution and placed in a thermostated holder at 5°C. The light source was a 1000 W DRSh-1000 high-pressure mercury lamp (Electromash, Chelyabinsk, Russia). The intensity of the light entering the cuvette, *I*_0_, was measured by ferrioxalate Hatchard-Parker actinometry [14] and found to be (1.43 ± 0.07) × 10^16^ photons/(s×cm^2^). Selection of excitation wavelengths was performed by using a BS-4 glass filter (LZOS, Lytkarino, Russia) which cuts off the light of wavelength lower than 340 nm. The high-pressure mercury lamp spectra consist of separate bands, and only one band (*λ*; = 365) over 340 nm can excite reagents because of its overlap with the Soret band of phthalocyanines. The concentration of the target in the reaction mixtures was 1 × 10^−8^ M, and the concentration of the conjugate 8.2 × 10^−5^ M. Such concentrations were chosen in order to force all oligonucleotide targets into a duplex with the conjugate. Irradiation of the samples in the visible spectrum region was carried out for 30 minutes in 1.5 mL tubes (Eppendorf, Wesseling-Berzdorf, Germany) using a helium-neon laser (633 nm, 10 mW) at 25°C. This wavelength corresponds to a Q-band of phthalocyanine absorption spectra.

The irradiated solutions were immediately transferred into polypropylene tubes containing 1 *μ*L of 1.4 mg/ml of total *E coli* tRNA; 400 *μ*L of 2% LiClO_4_ in acetone was then added. The precipitate was pelleted by centrifugation, washed twice with 80% ethanol and once with acetone, and dried in vacuum.

### Catalytic modification

Oxidative modification of the 22-nt oligonucleotide with the complementary oligonucleotide carrying a Co(II)-phthalocyanine moiety was performed at 25°C in two ways: (1) in the presence of H_2_O_2_ and (2) in the presence of O_2_ and 2-mercaptoethanol as a reducing agent. The concentration of the target in the reaction mixtures was 10^−8^ M, the concentration of the conjugate was 8.22 × 10^−5^ M, and the concentrations of H_2_O_2_ and 2-mercaptoethanol were 2.0 × 10^−4^ M. The concentration of O_2_ in aqueous solutions at 25°C at atmospheric pressure is 2.0 × 10^−4^ M. The sample volume was 10 *μ*L; the incubation time was 24 hours.

### Identification and separation of modification products

To cleave the modified oligonucleotide at the points of modification where it becomes labile to alkali treatment (apurinic/apyrimidinic sites and oxidized deoxyribose), the precipitates were dissolved in 50 *μ*L of 1 M piperidine (pH 12) and incubated for 45 minutes at 95°C [15]. The products were precipitated and washed twice with 95% ethanol, once with acetone, and then dried in vacuum.

To digest the product oxidized at deoxyguanosine residues, including 8-oxoguanine, which is relatively stable in alkali, the samples were treated with 8-oxoguanine-DNA glycosylase from *E coli* (Fpg protein). Prior to this treatment, the samples were washed twice with 85% ethanol and once with acetone to remove the buffer, and dried in vacuum. The precipitates were dissolved in 20 *μ*L of a buffer containing 50 mM Tris-HCl (pH 7.5), 50 mM KCl, 1 mM EDTA, 1 mM dithiothreitol, 9% glycerol, and 6.6×10^−6^ M Fpg. After the reaction, the enzyme was twice extracted by the mixture of phenol-chloroform-isoamyl alcohol (25 : 25 : 1 v/v/v). The reaction mixtures were precipitated with 10 volumes of 2% LiClO_4_ in acetone, centrifuged, washed twice with 95% ethanol, dried, and dissolved in a marker dyes solution containing 0.1% bromophenol blue and 0.1% xylene cyanol FF.

The products of modification were separated by 20% PAGE in the presence of 7 M urea. After electrophoresis, the gel was exposed to CP-BU X-ray film (Agfa-Gevaert, Mortsel, Belgium) for 10–20 hours at −10°C.

## RESULTS

The conjugates of deoxyribooligonucleotides with phthalocyanines of Al(III) and Zn(II) **I–IV** were studied as reagents for photochemical modification of nucleic acids (Figure 1). The oligonucleotides were connected to the phthalocyanine moieties through linkers with different numbers of CH_2_-groups: three in conjugates **I** and **III** and six in conjugates **II** and **IV**. Conjugate **V** containing Co(II)-phthalocyanine was used as a reagent for catalytic modification in the presence of molecular oxygen (Figure 1). The linker in this conjugate consists of three CH_2_-groups. A 22-nt oligonucleotide served as the DNA target. The structure of the complementary duplexes formed between the DNA target and the conjugates is presented in Figure 2.

### UV/Visible spectra of free phthalocyanines and conjugates

The conjugation of phthalocyanines with oligonucleotides was confirmed by the change in the spectrum shape at 600–700 nm in comparison with the absorption spectrum of free phthalocyanine. For free Co(II)-phthalocyanine, an absorption band was observed at 678 nm, with a shoulder around 630 nm. In the case of the oligonucleotide conjugate **V**, a band at 631 nm with a shoulder at 680 nm was detected [16]. For free Al(III)-phthalocyanine, the only absorption band in the visible region was observed at 678 nm; but for its oligonucleotide conjugates **III** and **IV**, two bands were found at 639 nm and 678 nm (see Figure 3). The spectrum of free Zn(II)-phthalocyanine contained a band at 643 nm with a shoulder at 680–700 nm (Figure 4). This band was broader than that for Co(II) and Al(III) complexes. The binding of Zn(II)-phthalocyanine with oligonucleotides **I** and **II** results in the appearance of a second band at 688 nm. All conjugates demonstrate absorption near 260 nm most probably due to the contribution of the oligonucleotide part.

### Sensitized modification under Hg lamp irradiation (*λ*; = 365 **nm**)

Modification of the 22-nt oligonucleotide was carried out at 5°C **.** When the reaction products were analyzed by electrophoresis in 20% PAAG, no direct cleavage of the target was observed. It is known that oxidation of DNA may lead to the appearance of alkali-labile sites [17]. These modifications are detected by the treatment of the reaction mixture with 1 M piperidine (pH 12) [15]. However, other modifications, which are stable in alkali, can also be found, for example, 8-oxoguanine. To detect this modification, the reaction mixtures were treated with Fpg protein, a repair enzyme from *E coli* that cleaves DNA at guanine residues oxidized at C8, as well as at some others oxidized guanosine derivatives [18].

**Figure 5:**
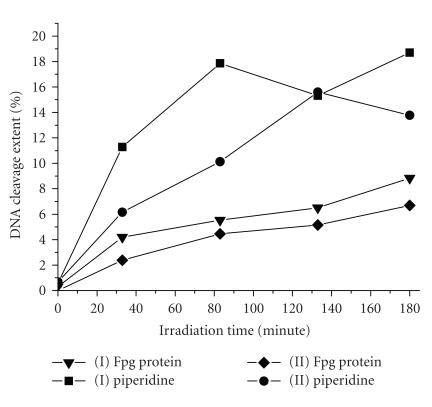
Time courses of photosensitized modification of the 22-nt oligonucleotide target with Zn(II) phthalocyanine conjugates **I** (▼, ■) and **II** (•, ◆) after irradiation with an Hg lamp at 365 nm. The postirradiation treatment was done using Fpg protein (▼, ◆) and 1 M piperidine (■, •).

The reaction yields of target modification by Zn(II)-phthalocyanine conjugates **I** and **II** containing aminopropanol and aminohexanol linkers were 18% and 14%, respectively, as detected by piperidine treatment (Figure 5). Treatment with Fpg revealed only 8% and 6% modification yield for conjugates **I** and **II**, respectively (Figure 5). A comparison of cleavage time courses demonstrates that the modification yield in the case of conjugate **I** is somewhat higher than for conjugate **II**. This may be explained by an increase in the probability of consumption of the active oxygen species in by-processes in solution due to a longer spacer of conjugate **II**. The longer spacer of conjugate **II** moves the phthalocyanine moiety farther away from the oligonucleotide target as compared with conjugate **I**, resulting in an increased contribution of by-processes with the solvent.

**Figure 6:**
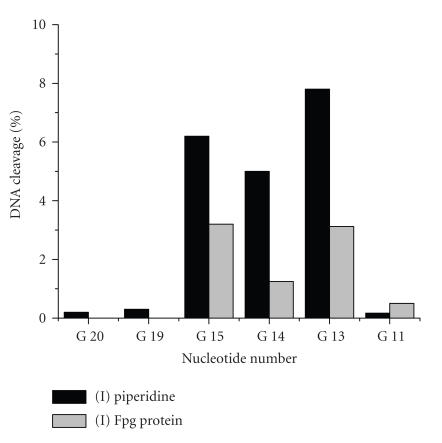
Distribution of base modifications in the 22-nt oligonucleotide target for sensitized modification with Zn(II)-phthalocyanine conjugate **I** after irradiation by an Hg lamp at 365 nm. The modifications were revealed by treatment with Fpg protein or 1 M piperidine.

**Figure 7:**
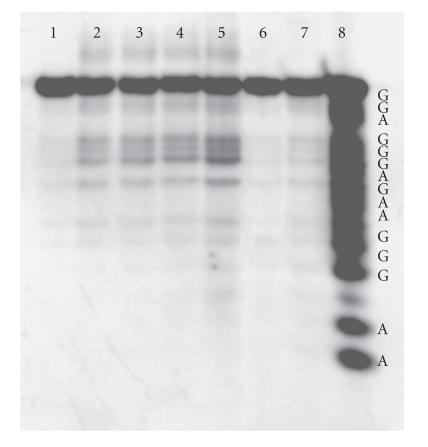
Autoradiogram of a 20% denaturing polyacrylamide gel showing a time course of cleavage of the 22-nt oligonucleotide target at 5°C by Zn(II)-phthalocyanine conjugate **I** after irradiation by an Hg lamp at 365 nm. Time points shown are 0 minutes (lane 1), 33.3 minutes (lane 2), 83.3 minutes (lane 3), 133.3 minutes (lane 4), and 180 minutes (lanes 5–7). The reaction mixture in lane 6 was not irradiated. The sample in lane 7 did not contain **I.** Lane 8 represents a Maxam-Gilbert (A + G) sequencing reaction.

The target modification by both Zn(II)-phthalocyanine conjugates **I** and **II** took place preferentially at guanine residues in the region G^11^–G^20^. Residues G^13^ and G^15^ were mainly modified, as can be seen from a histogram presented in Figure 6 for conjugate **I**. The autoradiogram of a gel corresponding to the time course of target modification by the conjugate **I** revealed by Fpg treatment is presented in Figure 7. The same data were obtained for conjugate **II**.

In the case of Al(III)-phthalocyanine containing conjugate **III** (data are not shown), the modification yield approached 12% as detected by piperidine treatment, and was close to 5% when Fpg protein was used to reveal modification points. The experiments with the Al(III)-containing conjugate **IV** resulted also in a rather low modification yield of 5–7% as revealed by both piperidine and Fpg protein treatments (data are not shown).

### Sensitized modification under helium-neon laser irradiation (*λ*; = 633 **nm**)

**Figure 8:**
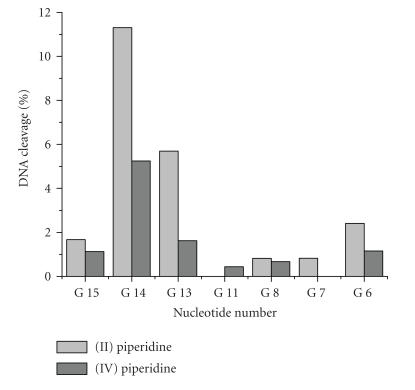
Distribution of base modifications in the 22-nt oligonucleotide target for sensitized modification with the Zn(II) and Al(III) phthalocyanines conjugates **II** and **IV** after laser light irradiation at 633 nm. The modifications were revealed by treatment with 1 M piperidine.

The reaction mixtures containing the phthalocyanine Zn(II) and Al(III) conjugates **II** and **IV**, respectively, were also irradiated by a helium-neon laser at a 633 nm wavelength for 30 minutes at 25°C. In contrast to the experiments with an Hg lamp, no cleavage of oligonucleotide target in the absence of these reagents was observed because biopolymers do not absorb light in the red visible range of a spectrum. The laser light was much more intense than the Hg lamp light, as can be seen from a significant decrease in the required irradiation time. The experiments have demonstrated (Figure 8) that the modification yield detected by piperidine treatment was higher for Zn(II)-phthalocyanine as compared with Al(III)-phthalocyanine and was 24% and 10%, respectively.

Distribution of modification sites for the phthalocyanine Al(III) **IV** and Zn(II)-phthalocyanine **II** conjugates were quite similar (Figure 8). In addition to G^13^–G^15^, noticeable cleavage takes place at G^6^, G^7^, and G^8^ bands. A comparison with the data obtained with an Hg lamp irradiation at 5°C suggests that such difference may be due to the higher flexibility of the phthalocyanine moiety and the higher mobility of the oxidizing species at higher temperature.

### Catalytic modification

The term “catalysis” used here applies to the mechanism of active oxygen species generation, where a metal-phthalocyanine moiety acts as a catalyst. The nucleic acid is oxidized by hydrogen peroxide, and metal-phthalocyanine catalyzes this process through reversible changes in its oxidation state.

Modification of DNA in the complexes with the Co(II)-phthalocyanine conjugate **V** were performed at 25°C with H_2_O_2_ and with O_2_ in the presence of 2-mercaptoethanol as a reducing compound. The PAGE separation of the reaction products has shown no direct cleavage of the target. Modifications were detected only after treatment of the reaction mixture either with 1 M piperidine or with Fpg.

The modification yield in the presence of O_2_ and 2-mercaptoethanol after 1 M piperidine treatment was found to be 15%, and in the presence of H_2_O_2_ it was approximately 35%. The highest modification yield was achieved in approximately 20 hours. The modification yields determined by Fpg protein treatment were significantly higher, 70% for the reaction mixture containing hydrogen peroxide and approximately 25% for the reaction mixture containing O_2_ and 2-mercaptoethanol.

**Figure 9:**
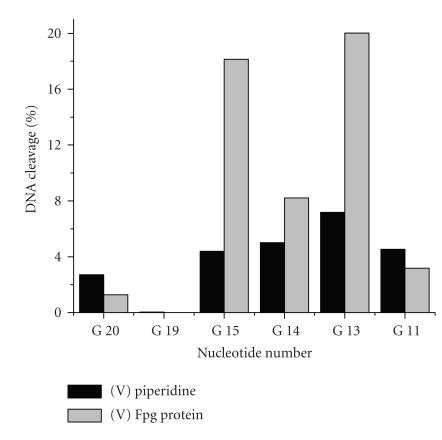
Distribution of base modifications in the 22-nt oligonucleotide target for catalytic oxidation by the Co(II)-phthalocyanine conjugate **V** in the presence of H_2_O_2_, after the modifications were revealed by treatment with Fpg protein or 1 M piperidine.

The target modifications were localized mainly in the G^13^–G^15^ sequence, similar to the situation observed with the Al(III)- and Zn(II)-phthalocyanine conjugates. The distribution of modifications was similar for the systems containing O_2_/2-mercaptoethanol and hydrogen peroxide (Figure 9). However, the depth of modification in the case of hydrogen peroxide was notably higher. This is not unexpected for a replacement of the system “O_2_ + reducing reagent” with H_2_O_2_, which is a direct source of hydroxyl radicals. Detection of modification points by Fpg protein treatment reveals a higher modification yield than does piperidine treatment, most probably due to a preferential modification at the C8 position of guanine.

## DISCUSSION

In the present work, we show that tetracarboxyphthalocyanines of Al(III), Zn(II), and Co(II) modify nucleic acids when delivered to their DNA targets by means of a complementary oligonucleotide tag, and that the modification can be initiated either by irradiation or in the reactions of catalytic redox cycling. Our experiments indicate that no direct cleavage occurs in the model oligonucleotides. Phthalocyanines presumably incur damage on different moieties in DNA depending on whether photosensitization or redox catalysis takes place. Apurinic/apyrimidinic sites and deoxyribose oxidation were revealed under alkaline conditions, and oxidized nucleotide bases like 8-oxoguanosine, by treatment of the samples with Fpg protein. It was found that in the presence of the Co(II)-phthalocyanine conjugate, the products of the catalytic modification in the DNA target are mainly substrates for Fpg protein but are poorly revealed by piperidine. When the high-pressure Hg lamp (365 nm wavelength) or laser irradiation (633 nm) were used, the sensitized modification products were better revealed by piperidine treatment than by Fpg protein. The relatively low levels of photomodification may be explained by the large migration distance (∼ 90 nm) of singlet molecular oxygen ^1^O_2_ along the polynucleotide chain compared to the 0.34 nm distance between DNA bases [19] or formation of modified bases of different structure, not revealed by the detection methods used.

Nevertheless, our results unambiguously demonstrate that modification of nucleic acids occurs when the target and metallophthalocyanine group are drawn together. Similar results in sensitized photomodification of nucleic acids were obtained earlier with conjugates containing other reactive groups, including porphyrins and their analogs, such as Pd(II)-coproporphyrin I [20], chlorine [21], sapphyrin, and Dy(III) texaphyrin [22]. Using oligonucleotide tags, it is possible to target the reagent to the required nucleic acid sequence, thus enhancing the selectivity of the process. Our results show that the generation of short-living oxygen species in the case of catalytic modification in the presence of the Co(II)-phthalocyanine reagent results in sufficiently effective and sequence-specific modification of nucleic acids. The reactivity of these reagents towards DNA or RNA of foreign organisms or malignant tumor cells raises the possibility of blocking the expression of specific genes and using phthalocyanine conjugates as drugs.
